# Book Reviews

**Published:** 1811-06

**Authors:** 


### CRITICAL ANALYSIS

OF

### RECENT PUBLICATIONS

IN THE

### DIFFERENT BRANCHES OF PHYSIC, SURGERY, AND MEDICAL PHILOSOPHY,

---

*Observations on Ophthalmia, and its Consequences.* By CHARLES FARRELL, M. D. 8vo. Lond. 1811. pp. 138. Murray.

DR. FARRELL arranges his work under "Introductory Remarks," and two Parts; the first containing three, and the second four Chapters: and its object is, to investigate the causes concurring to produce the ophthalmia which has appeared with such severity in the British army, to establish a division of this disease into varieties, and to institute a method of cure adapted to each variety.

Though instances of ophthalmia are to be met with in all places, and under every accidental circumstance of society, whether civil or military; yet the disease here noticed does not seem to have been known, at least in modern Europe, prior to the year 1801, or the landing of the British troops in Egypt. It is an observation made by our author, that the prevalence of ophthalmia in the British army followed the landing of the troops in that country; and that "Egypt is, perhaps, the part of the world, in which all the causes of ophthalmia, arising from soil and climate, exist in the greatest abundance and force." He considers the peculiarities of the soil and climate of Egypt as sufficient to account for the prevalence of the disease in that country; and he admits, or seems to admit, though he professes not to be able to determine how far, that this combination of causes may contribute to the production of a species of ophthalmia, which by frequency and inveteracy, "or other means," may acquire the power of propagating itself by *contagion*: and he sees nothing contrary to analogy in the supposition, that the violent species of ophthalmia, observed at present among our troops, is *contagious*. This fact he corroborates by the French army having suffered as much, or more, than ours, by this disease while in Egypt. The complaint accompanied this army to France, and is not, even yet, Dr. Farrell believes, eradicated. Here is the time and the place of its first appearance in the armies armies of Europe stated, the causes that combine in the soil and climate of Egypt to produce it, the probable reason of its becoming contagious deduced from its frequency and inveteracy arising out of those causes, and the actual fact asserted of its being disseminated among our troops by contagion; yet Dr. Farrell objects to its being denominated *Egyptian Ophthalmia*.

If we see no reason to reject the term Egyptian Ophthalmia, but rather to adopt it on the ground of Dr. Farrel's statement, we are not to be considered as advocates for the erroneous influence that names may produce. In this instance, however, we are not aware that this term has improperly directed the practice.

It is contended, and we believe with truth, that this disease, though it may be contagious, is inflammatory; and that its danger is in proportion to the violence of the inflammation. Nor does it appear that those practitioners who understand it best, and admit its contagious property, have been influenced in their treatment of it by the name; for the most active remedies employed against inflammation have been used to an extent in this ophthalmia, that would have been considered as bold to rashness, in any other disease.

"Though I have gone thus far," Dr. Farrel says, "in endeavouring to establish an Egyptian origin for the violent species of ophthalmia which has latterly excited so much attention, still I can see no reason why a violent inflammation of the eyes should not occasionally take place in other countries. I fear the fashion of putting down the ophthalmia observed in Egypt as a disease *sui generis*, and as considering it as different from every other inflammation of the eye, met with in Europe, has had some share in disturbing our opinions as to the true nature of the disease, and consequently leading us away from the right method of treating the disease."

With the author of these "Observations," we see no reason "why a violent inflammation of the eyes should not occasionally take place in other countries." We know that such a disease does occasionally take place, and we have had the misfortune to see it, in a most violent form, before the Egyptian expedition or Napoleon himself were thought of. But those cases, because they were only occasional and isolated, differed in some essential particular from that general affection of the eyes which afflicted our troops, as well as those of the French army in Egypt; and which was imported into Europe by the French and English armies, on their return.

Why a form of ophthalmia is endemial in Egypt, Dr. Farrell

Farrell states generally. The soil and climate of that country he considers as affording the causes for producing ophthalmia, in greater abundance and force than are found in any other part of the world. He believes that these causes have existed from remote antiquity. "All historians, both ancient and modern, who have given accounts of Egypt; and all medical men, both antient and modern, who have written on the diseases of Egypt, agree as to the frequency and violence of ophthalmia in that country." (Page 5.) If this be not strictly correct as to the antiquity of this ophthalmia, yet it is sufficiently substantiated that a disease of the eye, arising out of the preceding causes, has been known for several centuries. That the causes producing this ophthalmia did not operate in Egypt until that country became subject to the dominion of barbarians, is an opinion held by writers of great intelligence. And this is a fact of much importance, as shewing that the origin and propagation of the disease are connected with certain forms of society and habits of human life, conjointly with local peculiarities of climate; and therefore, in a degree, liable to the influence and controul of prophylactic regulations.

Under accidental circumstances of weather, perhaps a more than usual arid state of atmosphere, the prevalence and degree of some particular winds, and especially the increase and exasperation of diseases by war, may be induced an epidemic form of this endemial ophthalmia. Then it will happen that a disease, arising at first from external causes, becomes contagious, and propagates itself by a specific virus, either on direct contact with the sick, or in the more concentrated form of *fomites;* and this, when the remote causes no longer operate.

It is admitted that the ophthalmia here treated of, and which the most positive evidence does not permit us to doubt arose in Egypt, is contagious, and that its progress is in the course above stated. Whether it be a disease *sui generis*, having qualities peculiar to itself, specific and contagious *ab origine;* or whether the accumulation and concentration of morbid atoms, elicits or generates a contagious material, is not of much importance as relates to practice. If it is contagious, however acute, or however mild its inflammatory symptoms may be; or how much soever it may be proper to treat those symptoms on the general principles employed for subduing inflammation; the disease does NOT "resemble, *in every respect*, any other violent inflammation of the eyes, in which the climate or contagion of Egypt could have no share." (P. 7.)

It is here we object to Dr. Farrell's conclusions from his ov[illegible]

own premises. And we object, not so much because we think him wrong in his deduction abstractedly, but that we perceive it to confuse an important fact, and to influence, most seriously, the prophylactic practice. If practitioners are persuaded that the Egyptian ophthalmia does not differ in "*any respect*," from common cases of ophthalmia, however acute, no care will be taken to prevent its propagation by contagion. It will be seen on the slightest reflection, that this opinion is fraught with extreme danger. When we assume, not only on the unequivocal acknowledgment of Dr. Farrell, but on the general opinion as founded on known facts, and particularly on the investigations of Dr. Edmonston, that the disease hitherto known by the appellation EGYPTIAN OPHTHALMIA, is *contagious;* we cannot for a moment hesitate on the propriety of keeping that fact in view, and of reprobating every change in nomenclature that tends to confuse the disease with others of the same genus, which have no such property.

That names have often led to fallacious conclusions and erroneous practice we readily admit; and that the most sedate and cautious practitioners scrupulously refuse to designate diseases by determinate appellations, we have often witnessed. If this coyness, often arising from motives widely different from an attachment to scientific truth, in some instances may be proper, in others it certainly has an injurious result. The instance before us is of the latter kind. A rejection of the term *Egyptian* ophthalmia, will induce, indirectly perhaps, but it will induce a false security. Though the mode of treating individual cases is entitled to great regard, yet, as the disease is acknowledged to be of foreign growth, and is contagious, it is of still more importance to check its dissemination.

We have seen with regret an endeavour to establish an opinion, not original indeed, for it is found in obscure and almost forgotten authors, that some diseases, rapid in their progress, fatal in their termination, and spreading consternation and dismay wherever they appear, are not contagious; and that all precautions to prevent their introduction or restrain their propagation, are futile and absurd. Even in this opinion, much as it behoves legislative bodies and the guardians of public health to adopt it with fear and trembling, there is some consistency, because its authors deny the existence of the contagious principle. But Dr. Farrell admits, and indeed argues for the contagious quality in the Egyptian ophthalmia,* and yet concludes that, in no respect does it

* "The appearance, in the first British army that visited Egypt, of a more violent species of ophthalmia than had before, at least, been

 noticed differ from the common ophthalmia of Europe; and objects to an appropriate appellation connected with a distinction which he has shewn really to exist.

If the ophthalmia, which first appeared in the English and French armies, while they were in Egypt, is *contagious*, and those best informed on the subject admit it to be so, it differs essentially from the common inflammation of the eyes occurring in Europe before the year 1801, and as "observed among the people of Great Britain;" then the term *Egyptian ophthalmia* becomes appropriate, for it marks the origin of the disease, and connects with that origin its most formidable quality, CONTAGION: and the appellation, instead of disturbing our opinions, gives them a fixed point of rest, and impels to that caution which may restrain, perhaps, exterminate the disease.*

These remarks have arisen, not out of a propensity to censure, but from the importance of the subject, and a regard to our public duty. We should be much concerned to see the faculty, on such grounds, negligent of the only means that can prevent the dissemination of a most formidable disease; and that a separation of the infected from the healthy should be rejected, as futile and absurd.†

---

noticed by our army medical men; the degree to which the disease prevailed in the army; the continuance of it in some regiments after leaving the country; and the circumstance of its not only attacking a great number of men of the same corps after once appearing in it, but of its spreading from one corps to another; have *very justly* given rise to the opinion, that this *species of ophthalmia has been introduced into our army in Egypt, and that it is contagious.* In addition to these facts, I can safely state, that this *species* of ophthalmia prevailed, to an alarming degree, in some regiments in Egypt in 1807; and that it was much more prevalent for a year, or more, in some of the regiments which returned from that country to Sicily in that year, than in those which did not form a part of the expedition to Egypt. I can farther say, that when it made its appearance in any regiment, whether it had been in Egypt or not, it generally attacked a large proportion of the men of that corps. These are imposing facts; and though some of them may be accounted for without adopting the supposition of the disease being contagious, *still I am disposed to think that the impartial consideration of the whole of them will lead to, if not firmly establish the inference of the contagious nature of the disease.*" P. 51.

* "I have myself always acted on the supposition of the disease being contagious. Indeed were the evidence less strong and persuasive on this side of the question than it really is, I think it would be the duty of every medical man in the army to incline to it, until the matter is put beyond all doubt, as it holds out numerous and obvious advantages." *Farrell*, p. 53. Obs. &c. &c.

† We do not mean to state that Dr. Farrell considers the separation of the diseased from the healthy as useless. On the contrary (p. 54) he considers

Ophthalmia is considered in these "Observations," to be dividable, with practical utility, into three species. Two of these are described as acute, and one chronic. The acute species is subdivided into *O. mitis* and *O. gravis*. The 1st Chap. treating on *O. mitis*, we shall pass over with merely observing, that this variety of the disease is supposed to be a common affection of the organ of vision, occasioned by exposure to cold, &c. &c. &c. It readily subsided under the usual methods employed against local inflammations. This form of the disease Dr. F. found to be much more frequent in Sicily than in Egypt. Notwithstanding our author considers his *O. mitis* to be "that form of the disease with which most practitioners in private life are conversant," it appears to us to have been only his *O. gravis (Egyptian ophthalmia)* with a milder train of symptoms, arising in peculiar idiosyncracies, but more particularly meliorated by a removal from its parent climate. The *O. mitis* was sometimes met with in Egypt, but in Sicily it was the most frequent form of the disease. The chap. on *O. gravis* begins with observing, that "all the symptoms enumerated in the *O. mitis* are present in the *O. gravis*, but they exhibit in this latter a much greater degree of violence." In the 2d chapter, Dr. Farrell gives an animated, and, we fear, a faithful picture of this dreadful malady.

"The conjunctiva in the early stage of this disorder is red, swelled and turgid. The secretion of tears is copious. I have never met with a case in which this symptom was absent. The patient complains of excessive pain and roughness of the ball of his eye, and he cannot bear even a feeble light. The eye-lids are red at their edges, and swelled, and there is often a sense of weight and scalding of the eye. Sometimes the integuments of the forehead and temples are red, swelled, and painful; or there is a soreness of the integuments of the whole head, with head-ache, a hard quick pulse, rigors, and other symptoms of fever. In almost all cases there is pain shooting across the forehead or along the temples. To these symptoms succeed in a very short time, œdematous swelling and tension of the eye-lids, and prodigious tumefaction and turgescence of the conjunctiva, with a feeling as if the eye was to burst out of the head. If a ray of light now fall on the retina, the patient stoops his head with extraordinary celerity, covers his eye with his hand, and describes his feeling as if some sharp instrument was thrust into his eye, or as if something of a red colour darted through it. The eye-lids are painful to the touch, and the force necessary to use in opening them causes much distress. The coats of the eye being now enormously considers it of the utmost importance, and he has no doubt of diminishing the number of persons attacked, by thus cutting off the source of contagion. But if it is believed that this "resembles in every respect any other violent inflammation of the eyes," its specific character will be overlooked, and this essential species of quarantine neglected.

mously inflamed, distended and tense, and the eye-lids, and very probably the lachrymal glands, and every part contained within the orbit, being in a similar state, the globe of the eye must be greatly pressed, and its motion in the socket rendered difficult and painful. The muscles which move the eye, being themselves inflamed, are rendered highly irritable by this circumstance, and act with redoubled force on the ball of the eye. This violent action of the muscles is, no doubt, a source of pain in itself, as far as the muscles are concerned; but the violence of the action on the inflamed and distended ball of the eye, already pressed by the swollen parts within the orbit, must cause a lancinating pain in every motion of the globe of the eye. As the muscles of the eye are almost always put in motion by the impression of light upon the retina, on a person's coming from a dark place into the light, I think it probable that a portion of the excruciating pain following the impression of light on the retina in the case of violent inflammation, is owing to the violent action of the muscles on the ball of the eye, and to the difficulty of its motion in the orbit. In addition to all these causes of pain, it may be observed that the inflamed parts are chiefly membranous, or of an extremely delicate texture; and that inflammation in such parts runs a particular course, and is attended with most exquisite pain.

"In some cases the under eye-lids are turned outwards, in others both eye-lids are closed, and swollen, and the skin of these parts has an efflorescent, shining appearance. It is not unusual to see the eye-lids open, and the conjunctiva so much swelled and turgid as to protrude from the eye in the form of two or three folds. The tarsi in these cases are necessarily separated from one another, and the tumefied conjunctiva fills the whole anterior part of the space between the eye-lids, so that it is not possible to obtain, by any means, a sight of the cornea, or other parts of the globe of the eye. When, however, the tumefaction of the conjunctiva is not so great, and the eye can be brought fully into view, the cornea appears sometimes to be unusually pellucid; the pupil is contracted; the iris is discoloured, or, as it were, full of spots, and I have often thought that it was forced nearer to the cornea than it is observed to be in the healthy state of the eye."

This is but the first act of an awful tragedy, for as the inflammation proceeds,

"A secretion of a purulent-like matter takes place from the surface of the conjunctiva and glands of the tarsi. This matter is pent up for some time within the eye-lids, in cases in which the tarsi come in contact; and in others where these are separated, it flows out of the eye mixed with the tears. It is so acrid as to irritate the eye exceedingly, and to excoriate the palpebræ and cheeks in passing over them. The sufferings of the patient in this stage of the disease are excessive. He describes his feelings as if boiling water was poured into his eyes. He is hot and feverish. He can get no sleep by night or day; nor can he remain long in any one posture or situation. Bleeding from the temporal artery, large doses of opium, and washing the acrid matter repeatedly from the eye, will bring slight relief in most cases; but in others, no topical application or internal remedy will alleviate the sufferings of the patient, and he at last becomes delirious. If a sight of the ball of the eye c[illegible] be now obtained, it is found bathed with the purulent matter; a gre[illegible]

or depression, filled with this matter, runs round the whole circumference of the cornea; and the cornea itself is muddy in a part or the whole of its extent, or its surface is studded with small white spots. These appearances are unfortunately the commencement of a suppuration of the cornea, and when the disease has got this length, the muddiness partial or general of the cornea increases, or the white spots increase in size, or run into one another, and a suppuration of the cornea to a greater or less extent always takes place. Sometimes the whole of the cornea is included in the suppuration and destroyed; the iris is laid bare, the lens and vitreous humours are forced forwards on the iris, or entirely evacuated, and even the form of the eye does not remain. At other times, a portion only of the cornea suppurates, and the sight is more or less affected afterwards, according to the point at which the suppuration has taken place, and the extent to which it has gone. If the abscess be situated before the pupil, and if it penetrate the whole depth of the cornea, the aqueous humour, in escaping, will carry with it a portion of the iris through the aperture in the cornea, and the pupil will be generally totally obliterated by the protrusion of the iris, and its subsequent adhesion to the sides of the ruptured cornea."

The whole compages of the eye, locked within the orbit, and excessively increased in volume, occasion the most acute sufferings to the patient. From this painful state of compression he is relieved, however, by the coats of the eye giving way, generally, in a violent paroxysm of pain.

"In cases of partial suppurations of the cornea, the patient feels at the moment of the escape of the aqueous humour, as if something in his eye gave way, or as if fire was knocked out of it by a blow. The escape of the aqueous humour, and a protrusion of a portion of the iris, events apparently so much to be dreaded, are followed by an abatement of the sufferings of the patient. The tension of the eye, and of all parts within the orbit, and even that of the eye-lids, is considerably diminished by this circumstance. The inflammation of the eye begins to decline from this moment, and the state of the eye to improve, unless in the melancholy instances in which the iris continues to protrude."

It would occupy too many of our pages to follow Dr. Farrell through his elaborate, but, perhaps, irregular description of the train of morbid appearances arising in the progress of this disease; but we cannot help noticing at the close of his detail, that some doubts arise in his mind with regard to the rejection of the term Egyptian Ophthalmia. Though he has rejected this "title," he is "willing to allow, that if the frequency of a disease in any country gives a right to a special denomination, no name was *ever more justly applied.*"

In the treatment of this form of ophthalmia our author adopts the method of depletion, which has been carried to such a severe extent by other practitioners, particularly by Dr. Veitch.

"As

"As all the symptoms are of an highly inflammatory nature, and as the organ concerned can suffer no derangement in its structure, without greater or less injury to its functions, it is evident that a resolution of the inflammation must be the primary object. This, in a number of cases, is not to be accomplished, unless judicious and active means be employed within twenty-four or thirty hours after the accession of a violent degree of inflammation. Such is the tendency of the cornea to suppurate after this time, that the most active antiphlogistic means, pushed to a great extent, sometimes fail to prevent its suppuration. In no disease is it more necessary than in this to adopt a fixed and vigorous mode of treatment, in order to ensure success. As time is of the utmost importance, it will not answer to try first blisters and collyria, and then go on to blood-letting, topical or general—Bleeding, and that from one or both temporal arteries, as one or both eyes may be affected, to the amount of sixteen, twenty ounces of blood, or more, according to the urgency of the case and the strength of the patient, is to be the first measure. After this a large dose of salts is to be administered; the whole head is to be shaved; and large blisters are to be applied behind the ears and to the nape of the neck. The patient is to be lodged in a dark but well ventilated room, and the ball of the eye, if there be any purulent discharge, is to be repeatedly washed by injecting some mild fluid between the eye-lids. It is particularly necessary to avoid violence in injecting or introducing by any other means the fluid into the eye. I do not expect any advantage from this latter measure but that of simply removing the acrid and irritating matter from the surface of the conjunctiva. Whether there be a purulent discharge from the eye or not, the ball of the eye, palpebræ, and forehead, are to be kept constantly cool and moist, by means of one fold of fine old linen, wet with a weak solution of cerussa acetata, laid loosely over them. I must observe, that in some few instances the saturnine solution seemed to irritate and disagree very much with the eye; and that I have been obliged to desist from its use, and substitute for it a mixture of vinegar and water, or water alone. The cerussa acetata appeared to aggravate the symptoms in some cases in which there was a redness of the forehead and temples. When I had it in my power, I caused the temperature of the fluids I used in this way to be reduced by means of ice, and I applied them in that state to the eye."

The aggravation of the symptoms by the application of the solution of the superacetate of lead, as above noticed, is in some measure accounted for by the following remark:—"When this solution is used, it will be necessary to wash the linen occasionally with which it is applied, to prevent the accumulation of this preparation of lead on it, which happens in consequence of the evaporation of the water. By neglecting this precaution, it sometimes happens that a much stronger solution is applied to the eye than was intended." The observations upon collyria and other applications to the eye, as stated in our analysis of Mr. Stevenson's treatise, (p. 75.) will enforce the propriety of attending to this circumstance.

As

As the favourable termination of the disease uniformly depends on moderating the inflammatory action of the first stage, the subsequent observations in which the mode and degree of blood-letting are particularly detailed, must prove acceptable.

"If the inflammation do not become considerably more moderate in eight hours after the first bleeding, it will be necessary to take more blood from the temporal artery, regulating the quantity according to the intensity of the inflammation, and to apply a blister to a great part or the whole of the surface of the head. And if the inflammation has not completely subsided in eight hours more, it will be still necessary to repeat the bleeding from the temporal artery to a considerable amount if the symptoms seem to require it.

"In conformity to this plan, I have, in many cases, taken from the temporal artery from thirty to fifty ounces of blood in the space of the first twenty-four hours of the disease; and I have had the satisfaction to see the most violent degree of inflammation completely subdued by this practice in conjunction with the application of blisters and the other means just pointed out. It is not to be understood, however, that it is necessary to carry the bleeding in all cases to this extent, which I think may be fairly stated as the *maximum*. The inflammation, especially when attacked early, may be completely subdued, in many cases, by one bleeding of sixteen or twenty ounces, or by two bleedings of ten or fifteen ounces each, in conjunction with the effect of blisters and the other means. It is difficult to give precise rules as to the extent to which the bleeding is to be carried; but as it is the principal remedy upon which the cure of the disease rests, it is evident that it ought to be proportioned to the degree of violence of the symptoms, and repeated until all the inflammatory symptoms are very considerably abated."

To this powerful means of reducing inflammation, blistering to the head, to the neck, and behind the ears, purging, and a spare diet, were added as auxiliaries.

On the use of opium in inflammatory affections, opinion has been much unsettled. Conclusions formed on the hypothetical notion of its being a powerful stimulant, must necessarily condemn its employment in such cases: but actual observation has distinctly shewn, in a variety of instances, its useful effects, in opposition to the hypothesis. "I have no dread of this medicine," says Dr. Farrell, "at any moment of the inflammatory stage; but it is to be considered only as a palliative remedy. It is particularly well adapted to those cases in which the pain comes on in violent paroxysms."

The first and great object of the practitioner, in encountering this rapidly destructive disease, is, very early, to reduce the inflammatory action. If this cannot be effected, the structure of the visual organ will be destroyed, or so altered, as to impede, or to annihilate its functions. Frequent and copious bleeding seems the only means on which dependance can be placed. The abstraction of blood, according to the experience of Dr. Farrell, is only decidedly effectual when made from the temporal artery. Cupping, leeching, and bleeding from the arm, he considers as inadequate to this important object. As inflammation going on to a certain point, is always followed by a suppuration which destroys the organization of the eye, we cannot doubt the propriety of the energetic methods here laid down by the author.

Our limits will not permit us to go into a detail of the phenomena of the second stage of this disease, or those which arise when suppuration takes place. For the history and treatment of these we must refer to the work itself.

The third chapter treats of a form of Ophthalmia, which the author denominates *ophthalmia chronica*. The idiosyncrasies, or accidents that give rise to this variety of the disease, are thus stated.

"Ophthalmia is often found to pass into the chronic state in consequence of its repeatedly occurring in the same person. This state succeeds much more frequently to attacks of the virulent, than to those of the mild species of the disease. It sometimes assumes the chronic state after a first attack of the mild species; but this is in general owing to some peculiarity of constitution, or to bad treatment, especially the improper use of astringent or stimulant collyria. If one eye be weaker than the other, as is generally the case with persons who squint, the inflammation in the weak eye is extremely liable to pass into the chronic state, and to be afterwards revived by the slightest causes. As the other eye is affected by sympathy, persons with a defect of this kind, are liable to repeated attacks of inflammation in both eyes from causes which scarcely affect the eyes of others.

"I have found ophthalmia prone to pass into the chronic state, when it attacked patients labouring under other complaints, or persons in a state of debility recovering from disease. It sometimes assumes the chronic state in patients under the influence of mercury; and it is not unusual to see it pass into the chronic state in persons who have suffered much from pulmonic or rheumatic complaints, or in those labouring under herpetic affections, and in persons whose constitution is shattered by intemperance. It is extremely obstinate in all these cases, and often produces more or less imperfection of sight, or even total blindness."

A syphilitic taint of the system, without there being any affection of the bones of the orbit, has been adopted by Dr. Farrell, as one source of chronic ophthalmia. There have been doubts whether the syphilitic virus does ever produce inflammation of the eye, through the medium of its diffusion in the system. Mr. Hunter almost denies the occurrence of a symptomatic ophthalmia as arising from this cause. In this fluctuating state of opinion, we cannot refuse to lay before our readers the observations of Dr. Farrell, which appear pear to have been taken from nature, and to possess considerable discrimination.

"I have often suspected that chronic ophthalmia was connected with a venereal taint in the constitution. I was led to adopt this opinion, from having observed some peculiarity in the appearance of the eye and character of the inflammatory symptoms, when this form of disease occurred in persons labouring under secondary venereal symptoms, and more especially, when a venereal eruption on the skin formed one of them. Both eyes are generally affected; the patient complains little of pain, and still less of intolerance of light, and is not in fact conscious of any thing being wrong with his eyes, until the sight begins to grow dim. The eye is more watery than natural, but there is no great secretion of tears. The redness of the conjunctiva is not so vivid, as when the disease of the eye is unconnected with lues; the form of the pupil is sometimes irregular, or its edges are jagged, and the cornea is muddy in its whole extent, without any red vessels passing over it. Vision, as may be easily conceived, is totally lost for a time, or imperfect according to the degree of opacity of the cornea. This series of symptoms corresponds pretty well with the appearances of the eye in by far the greatest proportion of patients labouring under this complication of ophthalmia and lues; but the pain, weeping, and intolerance of light, go sometimes to a much higher degree. The inequality in the form, or jaggedness of the edges, of the pupil, will be found a pretty constant symptom; but I am apt to think, the opacity of the cornea, without any red vessels shooting over it, is the symptom the most strongly characteristic of a venereal taint being connected with the disease of the eye. There is in soldiers a peculiar expression of countenance, not easily to be described, which, in conjunction with the preceding symptoms, establishes in my mind the existence of a venereal taint in the constitution.

"The connexion between the affection of the eye and lues, is rendered still more probable, by the fact of the opacity of the cornea disappearing, and the redness of the conjunctiva vanishing as soon as the venereal symptoms on the skin, or in other parts, begin to disappear."

Many remedies and methods of treatment are suggested, as collyria, caustic, scarification, excision of portions of the conjunctiva, blisters, &c.; we can, however, for reasons before given, notice only in a general way, that our author found confinement in an hospital retarded the cure; that exposure to a moderated light was more serviceable than keeping the patient in darkness; and, that after every effort had failed to remove the symptoms, the disease frequently subsided in a short time, on discontinuing all applications to the eye.

The second part of the volume, on the "Consequences of Ophthalmia," is divided into four chapters, treating of Relapses, Ectropium, Entropium, and Pterigium.

In the chapter on Relapses is found the following statement

 of of the plan pursued in Sicily for the management of the convalescents from this disease.

"The convalescent, on being discharged from the hospital, is removed to a spacious well ventilated building, fitted up on purpose for the reception of such persons. Here he is given in charge to a military and medical officer, and he is in every respect as well accommodated and attended as he was in hospital. He is allowed his full ration of bread, meat, and wine, unless the medical officer see something in his case which will justity him in withholding the wine. As soon as he appears likely to be benefited by exercise, he is obliged to walk out in the open air, with a shade over his eyes, for such length of time as may be thought useful to him, under the command of a commissioned officer. The moment a relapse is discovered, and from the vigilance observed, such an event can exist only a short time without being detected, the patient is immediately sent back to the ophthalmic hospital, which, I may observe, is distant only a few hundred yards from the building occupied by the convalescents. But if all things go on well with the convalescent, and his eyes gain strength, he is selected out by a medical officer and transferred to another convalescent establishment which is some miles distant from the former. Here he is also comfortably accommodated and placed under the care and controul of a military and medical officer; but he is allowed to walk about a great part of the day at his own discretion, and he is occasionally exercised in marching, and other military employments. In this way his general health is improved, his eyes regain strength, and he is gradually habituated to exercise in the open air, and to the occupations in which he is to be afterwards employed. It is, among other things, the duty of the medical officer to see that the military discipline to which the convalescent is subjected be suited to the state of the eyes and general health. The convalescent is sent from this latter establishment to join his regiment, but not without being previously selected by a medical officer as a fit subject to be discharged.

"The advantages arising from this plan might be predicted from a knowledge of the disease and the habits of soldiers; and I have no hesitation in saying that they will more than equal a reasonable expectation Its good effects were not confined to patients purely convalescent. The same mode of management adopted with many patients in whom the disease had assumed the chronic state, and for whom little or nothing could be done in hospital, was found to be attended with manifest advantages, without the assistance of any topical remedy to the eye, or internal medicine. It will be often necessary, however, to contribute to the improvement of the eye by the ordinary remedies on such occa[illegible]ons, and to attend also to the means calculated to accelerate the reestablishment of the general health."

In works treating on the diseases of distant countries as observed on the spot by their authors, it does not unfrequently occur that the express subject of the volume makes its least interesting past. Incidental remarks on the manners, habits, and various peculiarities of the natives of remote countries; meteorological appearances; facts in the natural history of animals, animals, vegetables, and minerals, are often of great importance. Where these remarks and facts are recorded without a bias to system, from which the medical observer will seldom be free when treating of diseases, they become particularly valuable. Though the following relation (note, p. 48.) is not entirely free from hypothesis, we consider it as too curious to pass unnoticed.

"I often suspected (says Dr. F.) that the dust, carried about in such quanties by the winds in Alexandria, and in the adjoining desert, contained a considerable portion of pulverized salts. The surface of many parts of the desert abounds with salts, the water obtained by digging in almost every place about Alexandria, is brackish, and the ruins and walls of fhe houses of Alexandria are incrusted with saline productions. Perhaps some explanation of the fact of the salts being so abundant in the soil of Egypt, might be derived from the circumstance of the land being so nearly on a level with the sea. The formation of salts on the walls of the houses of Alexandria, and the absolute disintegration of the calcareous stone of which the walls are built, form an interesting subject of chemical inquiry. The stones have totally disappeared in parts of the walls of many houses, and the mortar in which they were bedded remains. It appears to me that the demolition of the stone is effected by its particles being detached from one another by the crystallization of the salts in its pores, and by a part of its composition going to the formation of saline compounds. Perhaps it is in the same way that the calcareous stones used for the construction of the ancient Syracuse, and many other cities, have disappeared, as it is said, from the face of the earth."

---

***Practical Observations on the Formation of an Artificial Pupil, in several deranged states of the Eye, to which are annexed Remarks on the Extraction of soft Cataracts, and those of the Membranous Kind through a Puncture in the Cornea. By*** BENJAMIN GIBSON, Surgeon to the Manchester Infirmary, &c. 8vo. Lond. Cadell and Davies, 1811. Plates. pp. 155.

SINCE the return of our army from the shores of Egypt, the attention of surgeons has been more particularly directed to the treatment of those derangements of the organs of sight, producing imperfect vision, than formerly; and which are frequently succeeding an attack of what is termed Egyptian ophthalmia, as well as the consequence of inflammation, induced by other causes. Anterior to that period our information was extremely limited, and, consequently, our operations were performed with hesitation and timidity. The experiments and writings of Daviel, Janin, Bart, Richter, Demours, Mounoir, Scarpa, and others, had completely established the pre-eminence of the Continental surgeons in this department department of Surgery. The former, though frequently unsuccessful, yet, being occasionally attended with advantage, justified their claim to superiority; whilst the subject was either miserably neglected, or only slightly investigated in this country. The present race of surgeons appear, however, to be fully aware of its importance; and the foundation of institutions for the reception of patients labouring under visual maladies, will, we trust, ere long, reclaim this neglected branch of surgical study from the obscurity in which it has hitherto been involved. This will be more especially the fortunate result, if competent talent be called in to support them. Impressed with this hope, and with the gratifying prospect held out, we hail the appearance of the volume before us with pleasure, as affording a valuable addition to the small collection of good works which have been written upon the subject. We shall endeavour to give our readers a brief analysis of its contents, in order to afford them some idea of the value of the observations it contains, and to incite them to peruse the whole at length.

In his preface Mr. Gibson takes the opportunity to remark, that some practitioners may be disposed to doubt the veracity of his statement respecting the extreme insensibility of the iris, from its generally having been supposed to possess the contrary properties in a superior degree. These opinions are now, however, proved to be founded in error. The experiments and operations of Professor Scarpa, and the extensive practice of Mr. Gibson, have enabled these gentlemen to assert, as an incontrovertible fact, that the muscular fibres of the iris are endowed with a very slight proportion of nervous influence, and are little disposed to inflame on being irritated. The knowledge of this fact is of the utmost importance, as the ignorance of it has hitherto deterred many surgeons from interfering with the more complicated disorganization of the eyes, under the idea that the most dreadful symptoms might be expected to supervene, in consequence of a wound inflicted upon the iris.

The first section of this work is entirely taken up with a concise history of the operation, as it has been performed by different surgeons with different variations; but the author avoids giving an opinion with regard to the relative advantages of each. Our countryman, Cheselden, appears to have been the first who devised and executed any operation with the intention of forming an artificial pupil. After him Sharp, Wentzel, Janin, Scarpa, Mounoir, Demours, &c. proposed operations to effect the same object, each of them varying from the others, but all being liable to objections in a greater or less degree, and not adapted to the various derangements rangements of the eyes requiring the operation under consideration. It has been Mr. Gibson's good fortune, by reasoning and experiment, to devise modes in order to supply the desideratum in question; and provide for every exigency. We shall proceed to a concise account of his operations, occasionally extracting from his volume.

"The mode of operation for forming an artificial pupil, it is obvious, must be varied according to the circumstances of the case. When there exists merely an opacity in the centre of the cornea without derangement in the internal parts of the eye; or, when the pupil is closed after the extraction, or depression of a cataract; in these cases the operation will be most simple. But if, along with central opacity of the cornea, the iris has formed adhesions to its inner surface, with, or without a corresponding diminution of the capacity of the anterior chamber of the aqueous humour, the operation becomes more complex. If again, along with opacity, and adhesions of the iris, the crystalline lens or its capsule be opaque, the adoption of still more complex expedients is necessary. The same may be observed of those cases, in which the pupil is totally obliterated, or nearly so, and the iris adheres to the capsule of the crystalline lens; for this rarely happens without an opacity of the lens or its capsule, or of both. Sometimes, however, a blow upon the sclerotic coat, particularly affecting the crystalline lens, and probably rupturing the edge of its capsule, is followed by an absorption of the lens, and leaves only the opaque capsule.

"In describing the operations, adapted to these cases, it is to be understood that the subjects of them were adults generally, who had either lost one eye, or had both eyes in similar circumstances of disease. I have never thought it of sufficient consequence to perform the operation where one eye was entire and perfect."

To accomplish the operation requisite to be performed under these circumstances, in addition to the cornea-knife and curved scissars, a small hook, a minute pair of iris-scissars, and a small pair of forceps, are necessary; the two last being of a peculiar construction, and devised by Mr. G. for the operation.

Opacity in the centre of the cornea is the first state requiring the operation which he treats upon. In this case he supposes at least a third part of the cornea to remain transparent and situated either at the superior or inferior part, internal or external angle of the eye. The latter spot he prefers, or towards the inferior part, as the operation is less difficult here.

"The first step of the operation is to secure the eyelids, as in the operation for extracting a cataract. A puncture is then to be made in the cornea with a broad cornea-knife, within a line of the sclerotica, to the extent of about three lines. All pressure is now to be removed from the eyeball, and the cornea-knife gently withdrawn. The consequence of this is that a portion of the aqueous humour escapes, and the iris iris falls into contact with the opening in the cornea, and closes it like a valve. A slight pressure must now be made on the superior and nasal part of the eyeball, with the fore and middle finger of the left hand, till at length, by an occasional and gentle increase of the pressure, or by varying its direction, the iris gradually protrudes, so as to present a bag of the size of a large pin's head. This protruded portion must be cut off with a pair of fine curved scissars, and all pressure at the same time removed. The iris will then recede within the eye, and the portion which has been removed will leave an artificial pupil more or less circular.

"It sometimes happens that the whole breadth of the iris, to the border of the natural pupil is protruded and removed in this way. This, I consider as rather an advantage, as it ensures a large pupil, though generally one which is oblong in its shape. I have found, however, the mere circumstances of shape to be of little consequence in this operation, and always to be sacrificed to the object of size. It may also be remarked that the opening has no disposition to close, when, in forming the artificial pupil, the border of the natural pupil is divided.

"It occasionally happens, also, that as soon as the knife is removed, the muscles of the eye-ball act with violence, and propel a small staphyloma or bag of the iris through the incision. If this bag be not large enough to form the new pupil, the iris must be further protruded by gentle pressure.

"As soon as the operation is finished, the eye is to be lightly covered with a few folds of fine linen, dipped in water, which may be cold in summer, and tepid in winter, and the patient is to be kept in an horizontal posture for a few hours. If pain should ensue, an opiate may be taken in the evening, and a saline purge the following morning. These medicines, however, are seldom requisite, but may be used as precautionary."

The puncture in the cornea ought not to be too large, and it is of importance to cut off the whole of the protruded portion of the iris with one stroke of the scissars; otherwise, on the escape of the aqueous humour, it recedes into the eye, and another opportunity for doing this, at the time, is not afforded. Should the artificial opening, as above mentioned, not appear sufficiently large to the operator, the iris may be drawn out of the eye, by means of the small hook, and divided, or (if it appear more easy) the iris scissars may be introduced into the eye, and the opening be enlarged, by cutting off a portion of the iris.

The subsequent inflammation in the eye is very slight. The aqueous humour is speedily regenerated (in an hour or two). Sometimes a slight cloudiness of this is perceptible, but the lapse of a few days serves to dissipate it. When the cornea is flatter than usual, and the wounded iris discharges, what may be termed, much blood, the case is rather unfavourable, the new pupil being much disposed to contract. This, however, may be prevented, we conceive, by the exhibition of the belladonna. The permanency of the newly formed formed pupil depends on its size, and the healthy state of the iris. If this be the medium size of the natural one, and, more especially, if it include its border, no contraction ensues; but, if the contrary to this be the case, an obliteration will occasionally take place. By means of this operation, a patient will be enabled to read a moderately small print, with properly adapted glasses. This new pupil does not possess the power of contracting and dilating, which the natural pupil has in an healthy state.

From the foregoing account, it will be evident that Mr. Gibson's operation has a decided superiority over all which have been hitherto recommended, as none of their projectors appear aware of the importance which attaches to leaving the capsule of the crystalline uninjured. We now know that the slightest wound inflicted upon this membrane will be succeeded by opacity, either of it, or of the lens: and, only one author who has written upon the subject has pointed out a mode, by which the risque may be avoided; but the operation is mentioned in very vague terms, its result not stated, and reasonable doubts may be entertained whether it was ever practised. "Ce n'est que d'après une tradition orale que notre illustre maître le Professeur Sabbatier a connu le procédé de Wentzel père. Cette habile oculiste incisoit la cornée dans les deux tiers inferieurs de sa circonference; il saississoit avec des pinces et soulevoit le centre de l'iris pour en exciser une portion dont le defaut laissoit une ouverture indélébils."* It does not appear that Sabbatier ever adopted this plan of operating†, and Wentzel describes a very difficult operation as the one which he and his father were in the habits of performing‡. No other comment in particular is made upon the above quoted account by the writer Leveille, than that a Surgeon of Angers had performed a similar operation, substituting a hook in lieu of the forceps.

The second operation which Mr. Gibson describes is one, adapted to those cases in which there is "central opacity of the cornea, attended with adhesions between the cornea and iris, which include only an inner portion of the border of the iris."

"The consequence of considerable adhesions existing between the iris and cornea, is, that the iris frequently cannot be protruded through an opening in the cornea to a sufficient extent to form, by its removal, an efficient artificial pupil, as in the operation already described; the

* Vide Scarpa sur les yeux, trans. François par Leveille, Ed. 2d. tome 2, p. 169.

† Vide Medecine Operatione, tome 3d.

‡ Vide Treatise on the Cataract, &c.

 attempt, attempt, however, may always be made, and the protruded part be cut off, however small it may be. If this expedient should not succeed, the next step must be to introduce the small hook though the part of the pupil, which does not adhere to the cornea, and to lay hold of the border of the iris. With a little care this membrane may generally be drawn out of the puncture in the cornea, and cut off, with the curved scissars. In doing this all laceration must be avoided." "Much, however, in these cases, must be left to the discretion and judgment of the operator. If, for instance, he should not observe any part of the iris, which can advantageously be drawn out with the hook, after making one attempt with that instrument, he will probably employ the iris-scissars, and enlarge the opening within the eye: or having clipped a portion of the iris, which now only adheres by a slight film, he will think the forceps the best means of removing it. Sometimes a slight point of adhesion between the iris and cornea may be advantageously separated by a cornea knife, at the time of making the puncture: or, the iris-scissars may be used for the same purpose, previous to using the hook; if it should be found impracticable to draw out the iris with the hook, on account of existing adhesions, the artificial pupil must be formed within the eye by the iris-scissars; which are to be used in the manner explained in the third operation."

Mr. Gibson's third operation is requisite in those cases where the adhesions formed between the anterior and inner edge of the iris, and the posterior surface of the lucid cornea, are very extensive, including the whole border of the iris, or nearly so. Here the attachments are to be partially divided by means of the cornea knife, at the time it is passed for the incision of the cornea. When this is effected, if the iris appear loose, the hook may be used to draw a portion of it out of the eye; but if this be not the case, the iris-scissars are to be introduced, and a portion of the membrane cut off.

We would recommend these two operations to the attentive perusal of our readers, the adhesion of the iris to the cornea being a frequent occurrence. It is often taking place in consequence of the evacuation of the aqueous humour through an ulcer which has penetrated the lamina of the cornea, producing irregularity and contraction of the pupil, which sometimes terminates in complete obliteration of sight.

Operation the fourth.—" Central opacity of the cornea, and total opacity of the crystalline lens, or its capsule; with or without adhesions of the iris to the cornea." This state of the eye is not of frequent occurrence, but when it is met with, a more complex and arduous operation is requisite, than any of the above. Mr. Gibson details the various steps necessary to be adopted, by relating an interesting case which our limits will not allow us to transcribe. In the first place, an artificial pupil was formed, as related above. The opake crystalline, and its capsule, were then broken down (after the lapse lapse of a few days) by means of Ney's couching needle, and portions of these impelled through the new pupil into the anterior chamber of the aqueous humour, in order to facilitate their solution and absorption. This not taking place rapidly, portions of them were evacuated through a small opening in the cornea, at two operations subsequently performed, and the remainder being absorbed, the pupil was left clear. The result of this case, which was as lamentable a one as is to be met with, where any chance remains of recovering the sight, completely evinces the talents and manual dexterity of the operator.

Our Author's fifth operation is to remedy the privation of sight in consequence of the total obliteration of the natural pupil, attended with an entire transparency of the cornea. This state is induced by inflammation of the iris, or internal parts of the eye, the result of blows, or other injuries done to it; and, is sometimes succeeding both the extraction and depression of the opake crystalline, or its capsule. In this case the lens may be present, or absent; adhesions may be formed between its capsule and the iris; and these, constituting its varieties, require different operations. When the lens is known to occupy its situation, it should be broken down or depressed with the needle.

"Then, the point of the cornea knife, after entering the anterior chamber of the eye, must be directed to that part of the iris, which is distant about one third of its diameter from the external angle. This is to limit the artificial pupil towards the outer part of the eye. Here, it is to be passed through the iris so as to make an opening equal in extent to about one third the diameter of that membrane. If it be found practicable, a smaller incision may be made opposite to this, by repassing the point of the knife through the iris, where the pupil is intended to terminate, at a corresponding distance from the inner angle. A flap may then, by a gentle motion of the cornea-knife downwards, be sometimes formed, and may be removed by the introduction of the iris-scissars. Generally, however, only the simple incision of the iris, without a flap, will be accomplished. Under such circumstances, one blade of the iris-scissars must be advanced through the incision in the iris, and the other between the cornea and iris, until they include about one third of its diameter. The iris must next be divided from the upper extremity of the incision, a little obliquely downwards, in such a way, that when the scissars are applied in a similar direction from the lower extremity of the incision in the iris, the new pupil may approach in figure to an equilateral triangle."

To each of the operations, Mr. Gibson has added cases in point extracted from a great number on which he has operated, not on account of their being more successful in their result than many others, but as they exemplify his various positions in a better manner. He has likewise added plates of the iris-scissars, and forceps, and views of the artificial pupils, both with and without opacity of the cornea. These last, though not done justice to by the engraver, shew, in a very accurate manner, the changes that ensue in the appearance of the eye subsequent to the operation.

Mr. Gibson closes his account of the artificial pupil with an enumeration of several varieties of blindness, in which the operation has failed. They are chiefly those cases in which the iris is more or less diseased, and altered in structure from its natural state.

The remaining part of Mr. Gibson's publication is occupied with the account of two operations for extracting the soft and capsular cataracts, through a puncture in the cornea. We regret that our limits will not allow us to detail, at length, these novel operations. The cataract in children is occasionally membranous, but mostly milky or pulpy. The capsule being tender at this early age, Mr. Gibson has adopted the plan of breaking it down with the couching needle, in which case it is always absorbed, and the patient restored to sight. Mr. Gibson has been in the habit of operating in this manner for the last ten years upon subjects of all ages. We are not aware that any other oculist (with the exception of the late Mr. Saunders) has put in practice this operation at so early a period of life. From six months to two years, Mr. G. prefers as the most eligible time for operating.

For an account of this mode of operating, in cases of capsular, along with descriptions of numerous varieties of this species of cataract, we must refer our readers to the volume itself. We shall, however, extract the relation of his operation in cases of soft cataracts.

"Every surgeon, who is accustomed to the operation of couching, cannot well fail to be aware, that he has a soft cataract to act upon, when he has felt the slight resistance which it presents to the couching needle, and observes how little change, in many cases, the instrument makes in its appearance. In these cases, when the removal of the cataract from the axis of vision cannot be effected, on account of its softness, it should be the aim of the operator to rupture, most freely, the anterior part of the capsule, and to break down the substance of the cataract, by passing the couching needle cautiously through it, in different directions, that the aqueous humour may more readily act upon it, and reduce it to a pulpy state. When this has been effected, and the eye has perfectly recovered from the operation, the surface of the cataract will probably have put on a flocculent appearance, and be fit for extraction.

"In performing this operation, the patient is to be seated and secured in the same manner as for the extraction of a cataract. The cornea-knife, of the largest size, is then to be introduced through the cornea towards towards the outer angle of the eye, at the usual distance from the sclerotic coat. If there should be any doubt of the free laceration of the anterior part of the capsule of the lens, the point of the cornea-knife should be directed obliquely through the pupil, so as to make a more free division of it. All pressure upon the eyeball must now be carefully avoided, and the cornea-knife gradually withdrawn, which is attended with the evacuation of a part of the aqueous humour, and some portion of the cataract. The curette is next to be introduced through the incision, and advanced towards the pupil, by which the whole of the cataract may commonly be removed by degrees in a pulpy state, so as to render the pupil perfectly clear. Its removal is generally much facilitated, by gentle pressure towards the vitreous humour, with the convex surface of the curette, whilst the point is inserted through the pupil. Sometimes, however, the cataract is not reduced to a sufficient degree of softness by the action of the aqueous humour; and this state makes its removal more slow, but seldom renders the repetition of the operation necessary; for, when a considerable portion of the cataract has been removed, the remainder is generally observed to be so much reduced in bulk, before the fit period for another operation, as to insure its speedy disappearing."

From these extracts our readers may form some idea of the value of the work from which they are taken. We have experienced much gratification in perusing it, and can strongly recommend it to the profession in general. Its appearance will form an important æra in the history of surgery relative to diseases of the eyes; and will fill up that chasm in British surgery which has hitherto, almost exclusively, been occupied by the productions of foreigners. Its external appearance does not bear any proportion to its internal merits; and the modesty of the former will surely put to the blush many professional writers who drain our pockets, and overload our critical board with their expensive and ponderous lucubrations. The volume before us is evidently the production of a man who has an ardent love for the profession; who has bestowed unwearied assiduity in gaining an insight into the knowledge of it; and, who has attained extraordinary excellence in its practical part.

